# Scars or Regeneration?—Dermal Fibroblasts as Drivers of Diverse Skin Wound Responses

**DOI:** 10.3390/ijms21020617

**Published:** 2020-01-17

**Authors:** Dongsheng Jiang, Yuval Rinkevich

**Affiliations:** Comprehensive Pneumology Center, Institute of Lung Biology and Disease, Helmholtz Zentrum München, Max-Lebsche-Platz 31, 81377 Munich, Germany; dongsheng.jiang@helmholtz-muenchen.de

**Keywords:** scarring, fibrosis, fibroblasts, regeneration, adhesion

## Abstract

Scarring and regeneration are two physiologically opposite endpoints to skin injuries, with mammals, including humans, typically healing wounds with fibrotic scars. We aim to provide an updated review on fibroblast heterogeneity as determinants of the scarring–regeneration continuum. We discuss fibroblast-centric mechanisms that dictate scarring–regeneration continua with a focus on intercellular and cell–matrix adhesion. Improved understanding of fibroblast lineage-specific mechanisms and how they determine scar severity will ultimately allow for the development of antiscarring therapies and the promotion of tissue regeneration.

## 1. Introduction

The most common response to injury in mammalian tissues and organs is scarring and fibrosis [1]. The more favorable outcome would be complete regeneration, such as occurs in invertebrates and lower vertebrates [2], with new tissue retaining the same structural, aesthetic, and functional attributes as the original uninjured tissue. Mammalian skin exhibits a remarkable diversity of scar severities that depend on the injury type, anatomic location, age, gender, and species, and this kaleidoscope of responses includes cases, albeit rare in mammals, of scarless regeneration. In the back-skin, scarless skin regeneration is conserved in early mammalian embryos, but it is lost in humans before the third trimester (week 24 of human gestation) and in rodents around embryonic day 16.5–18.5 [3].

A multitude of therapies have been introduced to treat and/or prevent skin scars, but the efficacy of commercially available therapies remains limited [4], largely due to lack of a comprehensive understanding of the underlying cellular and molecular mechanisms that lead to scar formation. Scarring is most noticeable in the skin, but it affects almost all adult mammalian and human tissues and organs [5].

In this review, we summarize recent findings on the skin’s response to injury, with a focus on “decision-making” mechanisms of fibroblasts in injured skin that dictate the skin’s wound response to scar or regenerate.

## 2. Fibroblast Origins and Heterogeneity

The most prominent feature of fibrotic scars is the accumulation of an abnormally organized lattice organization of the extracellular matrix (ECM), which replaces the original porous lattice organization that characterizes healthy skin tissues. The archetypal cells that deposit and reorient the extracellular matrix, and which directly impose either scarring or scarless outcomes to injured connective tissues, are fibroblasts. Several surface molecules have been commonly used in the field as pan fibroblast markers, such as platelet-derived growth factor (PDGF) receptors (PDGFRα and PDGFRβ) [6,7], CD90 (Cluster of Differentiation 90 or Thy-1) [8,9], fibroblast-specific protein 1 (FSP-1 or S100A4) [10], and vimentin [11]. However, none of these markers is specific or broad enough for the entire fibroblast population. More importantly, earlier studies by us and by others suggest that scars are intrinsic cellular properties of a distinct fibroblast cell lineage (or population subset) [12,13]. The behaviors of individual fibroblastic populations determine the extent of fibrotic outcomes. Below we summarize various dermal fibroblast subsets that have been identified in recent years as contributing to scar formation (Table 1).

### 2.1. Fibrogenic Fibroblasts Are Defined by Function

Fibroblast subsets exhibit unique functions, on the basis of which they can be defined and classified. For example, by using genetic lineage tracing approaches of embryonic markers, such as Engrailed-1 (En1) and Wnt Family Member 1 (Wnt1), we have discovered several lineages of fibroblasts that inhabit either distinct or overlapping skin locations. Two such lineages co-inhabit murine back-skin: En1 lineage positive and negative fibroblasts (EPFs and ENFs, respectively). While EPFs are the primary contributors to all fibrotic outcomes after wounding, UV irradiation, or melanoma tumor formation, ENFs, on the other hand, do not contribute to fibrotic outcomes, but are the primary fibroblast cell lineage which forms the dermis during embryogenesis [13]. Further, we have shown that the back-skin undergoes a change in fibroblast cell lineage composition from ENF-predominant to EPF-predominant during skin development. The succession of these two fibroblast lineages leads to a phenotypic shift in the skin’s response to injury, from fetal regeneration to adult scarring [14]. Similarly, by genetic fate mapping, Dulauroy and colleagues have shown that transient expression of a disintegrin and metalloprotease domain 12 (ADAM12) identifies a subset of perivascular PDGFRα^+^ fibroblasts that overproduce collagen during scarring in dermis and muscle [16]. Recently, a small population of Paired Related Homeobox 1 (Prrx1) enhancer-positive (Prrx1^enh+^) fibroblasts located within the subcutaneous compartment that includes fascia and adipose tissue was shown to expand robustly upon skin wounding and contribute to healing tissues [15]. Similarly, the activation of the c-Jun–PI3K–AKT pathway is required for the rapid proliferation and migration of alpha-Smooth Muscle Actin (α-SMA)^+^ fibroblasts [17], and sustained nuclear expression of c-Jun has been documented in various organ fibrosis and can be used to define a fibrogenic fibroblast subset.

There are additional markers that are used to define fibrogenic fibroblast lineages. However, most lineage markers (such as En1, ADAM12, and Prrx1 enhancer) are either transcription factors that have a narrow time window for expression or intracellular enzymes that are not directly applicable for enrichment purposes. Therefore, these markers are not immediately pertinent for defining fibroblastic populations.

### 2.2. Fibrogenic Fibroblast Functional Subsets Defined by Spatial Location and Surface Markers

Dermal fibroblast subsets have been historically defined by their spatial location within either the upper papillary dermis (PD) or lower reticular dermal (RD) compartments. Driskell and colleagues have shown that in the developing mouse back-skin, the Leucine-rich repeats and immunoglobulin-like domains protein 1 (Lrig1)^+^ PD fibroblasts are required to induce hair follicle formation during skin development, whereas Delta like non-canonical Notch ligand 1 (Dlk1)^+^ RD fibroblasts produce ECM and are responsible for dermal repair and fibrosis [12]. The same group later indicated that the functional differences were due to distinct epidermal instructions. Upon skin injury, PD fibroblasts respond to epidermal Wnt/β-catenin and downstream Sonic Hedgehog (Shh) signaling, whereas RD fibroblasts respond to epidermal transforming growth factor beta (TGFβ) signaling by undergoing cell proliferation and secreting ECM [18].

The concept of a spatial restriction of functionally distinct fibroblast subsets is further supported in human studies. A follow-up study by the same group found functionally distinct dermal fibroblast populations present in human breast skin. A CD39^+^CD90^+^ fibroblast population was enriched in upper dermal compartments and supported epithelial differentiation, including rete ridge formation. A separate CD36^+^CD90^+^ fibroblast population was found to be enriched in lower dermal compartments [19] and involved to a larger extent in scar formation. Similarly, the fibroblast activation protein (FAP)^-^CD90^+^ population has been suggested to enrich for reticular fibroblasts, but they are not restricted only in the lower dermis [20]. To date, none of these surface markers have been able to unambiguously define the fibrotic subpopulations across all skin locations [9]. A defining marker that allows purification of fibrogenic fibroblasts across skin locations and age still remains to be determined in both mouse and human skin.

A strategy that combines genetic lineage tracing with surface markers may improve the classification of fibroblast subsets. Recently, Shook and colleagues showed that the predominant population of activated myofibroblasts in the wound bed expresses a Sca1^+^CD34^+^CD29^+^ cell surface profile. These cells appear to be derived and are a subset of the previously characterized EPFs [21]. In adult mouse skin, over 90% of EPFs express CD26 (dipeptidyl peptidase-4, DPP4) [13], which is a cell-surface serine protease that cleaves X-proline dipeptides from the N-terminus of polypeptides. Therefore, CD26 could be used as a surrogate marker to purify EPFs. The CD26^+^Lin^-^ fibroblasts have been demonstrated to be responsible for scarring after wounding, radiation fibrosis, and melanoma stroma formation [13,22], wherein diprotin A, a CD26 inhibitor, leads to decreased scarring [13]. Fibroblast activation protein (FAP) is another member of the same subfamily of serine integral membrane proteases (SIMP) and is closely related (50% identity) to CD26 [23]. Both CD26 and FAP have been shown to be associated with keloid scars [24,25]. Therefore, a combination of surface markers Sca1, CD34, and CD29, or CD26 or FAP alone, could be used as molecular targets for fibroblasts within the fibrotic EPF lineage. Such marker classifications in human dermal fibroblasts are still incomplete and wait to be determined.

### 2.3. Fibroblastic Cell Plasticity

Increasing new evidence supports the idea that scarring could be reduced by manipulating the plasticity of fibroblasts.

By using an in vitro 3D microtissue scaffold cleft model, Kollmannsberger and colleagues demonstrated that the fibroblast-to-myofibroblast transition is reversible and is controlled by tensile forces, possibly via nuclear translocation of Yes-associated protein (YAP) [26]. YAP is a key component of the Hippo signaling pathway that typically responds to mechanical strains and changes in cell–cell and cell–ECM adhesions [27,28,29]. Notably, the myofibroblast phenotype can be sustained in the presence of YAP by tensile forces, and in the absence of TGFβ. In addition, the fibroblast-to-myofibroblast transition has been reported to be mediated by EGFR signaling in dermal fibroblasts. EGFR ligands such as TGF-α and heparin-binding EGF-like growth factor (HB-EGF) are present during all stages of wound repair [30]. Co-localization of EGFR and hyaluronan receptor CD44 within membrane-bound lipid rafts induces MAPK/ERK, followed by Ca^2+^/calmodulin-dependent protein kinase II (CaMKII) activation, leading to myofibroblast activation and collagen deposition [31]. Aberrant persistence of EGFR signaling can lead to hypertrophic scars or keloid scars [31,32], partially due to paracrine upregulation of EGFR on fibroblasts [33]. Aged dermal fibroblasts exhibit reduced EGFR expression, and this decline correlates with decreased responsiveness to EGFR mitogenic signaling, manifested in delayed healing and reduced scarring [30,34].

During wound healing, myofibroblasts have been demonstrated to display morphologic similarities and marker gene expression resembling that of adipocytes, and this phenotypic change from a myofibroblast towards adipocytes is associated with reduced fibrosis and with formation of adipocyte clusters around hair follicles [35]. A similar lineage relationship between fibroblasts and adipocytes has been reported in the lung. Here, fibroblasts appear to display a phenotypic plasticity towards lipogenic and myogenic lung fibroblasts. A transition towards a myogenic contractile lung fibroblast determines fibrosis formation, whereas a transition towards lipogenic cells is seen during resolution of fibrosis, and this phenotypic transition is reported to be reversible [36].

A recent study using single-cell RNA sequencing analysis on mouse skin indicated that a subset of wound myofibroblasts express myeloid lineage markers, such as Lyz2 (LysM), CD34, and CD14; hence, the authors concluded that wound myofibroblasts may be derived from leukocytes [37]. A similar concept has also been proposed by Sinha and colleagues, who discovered that about two-thirds of myofibroblasts in healing wounds may be derived from myeloid cells. The authors claim that a conversion of macrophages into fibroblasts occurs during wound healing and requires instructions from keratinocytes through the release of extracellular vesicles (EV) containing microRNA-21 [38]. The concept of leukocyte-to-fibroblast transition was initially raised 25 years ago, and these leukocytes were termed as fibrocytes [39], with altered metabolic activity that favors oxidative phosphorylation [40].

In all of the abovementioned studies, the definitions of fibroblasts, myofibroblasts, adipocytes, and myeloid cells all depend on the expression of selected markers, rather than functional criteria. In our view, the documented transitions could very well reflect changes in cell morphology and marker gene expression in response to microenvironmental challenges upon wounding. Further investigation on the functional identity of such “fate-switched” cells is required to determine whether such transition involves genetic transdifferentiation/de-differentiation events or merely reflects dynamic change in phenotypic markers.

## 3. Mechanisms of Skin Scarring

### 3.1. Granulation Tissue and Myofibroblasts

The currently accepted view of scarring is that wound healing and subsequent scar formation is mediated through granulation tissue formation and myofibroblast differentiation at sites of granulation tissue. TGF-β released from wound inflammatory cells at sites of granulation tissue induces the transition of fibroblasts to contractile myofibroblasts. These myofibroblasts, together with their deposited ECM and blood vessels, form the granulation tissue that is responsible for wound contraction and new collagen deposition at the sites of wounds [41]. This concept has been adequately summarized in previous reviews [42,43]. The “granulation tissue” model stipulates the following four tenets: (1) fibroblasts locally reprogram into contractile myofibroblasts; (2) reprogramming occurs at sites of granulation tissue; (3) scars are locally deposited by fibroblasts interacting locally with granulation tissue; and (4) tissue contractures occur through fibroblasts pulling on granulation tissue itself. This view stems from morphological observations and from in vitro studies that mimic the granulation tissue environment, by seeding fibroblasts on various fiber matrixes. The matrix remodeling by the fibroblasts led to the prevailing conclusion that granulation tissue is the key element in the generation of scars at the wound center, and therefore, the cross-talk between fibroblasts and their matrix is essential for wound contraction and closure [44,45].

### 3.2. Cell–ECM Adhesion

In the granulation tissue model, the contraction force generated by myofibroblasts pulling on granulation tissue largely relies on cell–ECM adhesion. This notion is supported by the observation that human skin wounds with greater tension often form more extensive scars than those experiencing minimal tension [46]. Studies on mechanotransduction indicate that fibroblasts sense microenvironmental tension and convert mechanical forces into biological signals, which in turn regulate the fibrotic responses [5]. For example, activation of focal adhesion kinase (FAK) in fibroblasts results in increased scar formation [47], whereas placing tape on wounded skin to reduce tension has been shown to lead to reduction in the severity of skin scars.

Cell–matrix adhesion mediated by the integrin family plays a dominant role in this process via the integrin–FAK–Rho GTPases pathway [48]. Traction force microscopy and time-lapse imaging on 3D fibroblast cultures have shown that wound closure is driven by fibroblastic migrations controlled by mechanical forces at the wound edge, and minimally by fibroblast proliferation [48]. Blocking α_v_ integrins on PDGFRβ-expressing fibroblasts attenuates tissue fibrosis [49]. Reed and colleagues have pinpointed that α_v_β_1_ integrin mediates TGFβ1 activation by directly binding to the latency-associated peptide of TGFβ1, thereby promoting a fibrotic response [50]. In addition, integrin complexes at cell–ECM focal adhesion sites activate the Hippo pathway and downstream YAP/TAZ, changing the actomyosin cytoskeleton in response to changes in the ECM’s physical properties [51,52]. The underlying mechanism is not fully understood and has been suggested to occur through the modulation of nuclear pores [53] or through tension sensed on perinuclear actin caps [54] that influences YAP/TAZ trafficking into the nuclei. Persistent nuclear expression of YAP/TAZ in fibroblasts leads to their mechanoactivation on the ECM that amplifies and sustains fibrosis [55].

### 3.3. Cell–Cell Adhesion

The tension in wounded tissue is not only attributed to the tensile forces between fibroblasts and the ECM, but also significantly contributed by cellular forces exerted among activated fibroblasts in a cooperative manner. Accumulating evidence suggests that cell–cell adhesion between fibroblasts is as important as cell–matrix adhesion in wound contraction and scar development.

For example, cadherins are a family of calcium-dependent cellular adhesion molecules that form adherens junctions to bind cells with each other [56]. Mesenchymal cadherins such as N-cadherin (cadherin-2, CDH2) and OB-Cadherin (cadherin-11, CDH11) are expressed on fibroblasts [57]. The expression of CDH11 on dermal fibroblasts is elevated in the skin of patients suffering from systemic sclerosis or scleroderma [58,59], which are chronic autoimmune diseases clinically manifesting as progressive fibrosis of the skin and internal organs. CDH11-knockout mice showed attenuated bleomycin-induced dermal fibrosis, as quantified by measurements of skin thickness, collagen levels, myofibroblast accumulation, and profibrotic gene expression [59]. Furthermore, inhibitory anti-CDH11 monoclonal antibody decreased dermal thickness and fibrotic mediators in the bleomycin-induced dermal fibrosis model [59] and in a mouse model of systemic sclerosis called the tight-skin mouse [60]. In vitro studies mechanistically demonstrated that CDH11 regulated the migration of linked fibroblasts and the production of TGFβ from linked macrophages [59,61]. Another possible mechanism of cadherins involved in fibrosis is via Wnt/β-catenin signaling. Catenins are important components in adherens junctions that link the actin cytoskeleton with cadherins and help to transmit contractile forces into nuclear signal transduction [56]. Although there is ongoing debate on how cadherin expression affects the intracellular pool of catenins, studies on epithelial–mesenchymal transition suggest that cadherins are required for augmented activation of the Wnt/β-catenin pathway in vivo [62,63]. It is tempting to speculate that transient elevated CDH2 and CDH11 expression on fibroblasts in response to skin injury leads to augmented Wnt/β-catenin signaling, subsequently relayed to the activation of mechanistic target of rapamycin complex 1 (mTORC1) in fibroblasts, and thus induces the scarring program [64].

Similar to CDH11, intercellular adhesion molecular 1 (ICAM-1, CD54) expressed on fibroblasts has also been shown to contribute to skin fibrosis in the tight-skin mouse, since ICAM-1 deficiency or knockdown by antisense oligonucleotides significantly suppressed development of skin sclerosis [65]. ICAM-1 on fibroblasts is able to bind to integrins expressed on infiltrated leukocytes, such as T cells [65] and mast cells [66], and results in activation of both fibroblasts and adhered leukocytes, amplifying the fibrotic response.

Gap junctions are connexin channels formed between cell membranes that enable direct cytoplasmic coupling between cells, permitting intercellular passage of ions and small molecules such as Ca^2+^, ATP, and cAMP [67,68]. The major connexin expressed in mammalian skin is Connexin-43 (Cx43) [67], which has been shown to regulate and orchestrate fibroblast cellular adhesion and communication; thus, Cx43 is involved in wound healing and scar formation [69]. Cx43 deficiency or downregulation using antisense oligonucleotides accelerated wound closure in mice [70,71] and attenuated a rat model of diabetic wounds [72]. Intriguingly, Cx43 promotes scar formation, and its fibrotic actions may be attributed to its transcriptional activation of CDH2 [73]. The determination of the subcellular localization of Cx43 in wound fibroblasts would help to dissect the exact function of Cx43.

### 3.4. Alternative Model

The notion that granulation tissue mediates wound contracture and scar formation has already been challenged in experiments documenting wound closure after repeated removal of granulation tissue from wounds [74]. Moreover, the granulation tissue notion fails to adequately explain the disparate responses to injuries (varied degrees of scarring and contracture) of skin across anatomic locations and developmental stages. For example, an injured oral cavity is regenerated with minimum scars or contractures, despite granulation tissue forming.

An alternative model was originally proposed in 1950s. In this model, wound healing and scar formation is driven by wound contraction that happens at the wound edge, and the central granulation tissue is not required [75,76]. The abovementioned recent studies on fibroblastic cell adhesion favors the idea that wound closure and subsequent scarring is mediated by exclusively relocating pre-committed myofibroblasts into sites of wounds. Thus, cell-to-cell contact, rather than cell-to-matrix, could facilitate fibroblasts at wound edges to pull the intact tissue inwards by directional collective migration towards the center of the wound. This model is in line with the observation that fibroblast-to-myofibroblast transition is driven by cellular forces at the wound edge in a 3D engineered cleft model [26]. Intriguingly, in this model, the myofibroblast phenotype is stabilized at the growth front, even in the absence of endogenously supplemented TGF-β, and reverts back to a quiescent fibroblast phenotype already 10 µm behind the growth front [26]. Similarly, by traction force microscopy and time-lapse imaging, Sakar and colleagues showed that wound closure is driven by tissue-level deformation followed by fibroblasts tangentially migrating along the wound edge controlled by mechanical force, but not by fibroblast proliferation [48]. Both studies imply that the central granulation tissue is not necessary for wound-edge-driven contraction. The answer to which model is closer to physiologic settings awaits new insights from live imaging studies that can cover the entire process of wound healing and scarring.

## 4. Mechanisms of Scarless Healing

### 4.1. Regeneration across Developmental Age

In mammals, skin injuries that are inflicted to fetal skin at gestational stages earlier than the third trimester of pregnancy (or E16.5 in mice) result in scar-free healing and regeneration [77,78]. Scarless regeneration is usually seen across lower vertebrate taxon groups and appears to be present in mammals during early fetal life, but is then lost between late fetal and early newborn stages.

Several differences between embryonic and adult skin have been considered to drive this regeneration-to-scarring phenotypic transition. Weaker immune responses are found in fetal wounds, including low numbers of neutrophils, macrophages, and mast cells, and low levels of proinflammatory cytokines IL-6 and IL-8 [79]. The innate immune cells, including neutrophils and macrophages, are one of the important constituents of the skin wound microenvironment. Their direct and indirect interactions with dermal fibroblasts undoubtedly influence the cellular behaviors of fibroblasts during wound healing. A few recent reviews have excellently summarized and discussed the functions of macrophages and neutrophils in scarring and regeneration with molecular details [79,80,81,82,83,84]. On the other hand, different immunodeficient lines, such as Rag-deficient mice, severe-combined immunodeficient (SCID) mice, athymic mice, and immunosuppressant (cyclosporine A) treated mice, all respond to skin injuries with scars, indicating that the adaptive immune system plays no major role in dictating regeneration versus scarring outcomes in skin wounds [85]. The ratio between metalloproteinases (MMP) and their inhibitors (TIMP) and the ratios between the anti-fibrotic cytokine TGFβ3 and the pro-fibrotic cytokine TGFβ1 appear higher in fetal compared to adult skin [86,87]. However, TGFβ1 antagonists are ineffective in reducing human scars, arguing that these differences are not causative factors of the regeneration-to-scarring transition.

We recently discovered that during embryonic skin development, En1 lineage negative fibroblasts (ENFs), which minimally contribute to scar formation in adult wounds, are the dominant population in the early developing dermis [13]. Further, we found that the ratio of ENFs to EPFs (the predominant fibroblastic lineage contributing to scarring in adult wounds) decreases during development, wherein EPFs succeed in forming the majority between E16.5 and E18.5 [14]. By transplanting purified ENFs and EPFs separately into donor wounds, we showed that the change in fibroblast lineage composition from ENFs to EPFs during development leads to the phenotypic transition from regeneration to scarring [14]. Still, the fibroblast lineage-specific molecular mechanisms that instruct scarring versus scarless regeneration in response to injury remain undisclosed.

Aging also affects skin fibrosis. Under conditions of optimal wound care, in elderly patients, wounds heal with reduced scars [88]. Recent studies have shed some light on the cellular mechanism of this phenomenon. By single-cell transcriptomics analysis and long-term lineage tracing assay, researchers have shown that the lineage heterogeneity and functional and spatial identities of adult dermal fibroblasts are progressively lost during aging [89,90]. Nishiguchi and colleagues demonstrated in aged mice an astonishing full-thickness tissue and hair follicle regeneration of ear punches and diminished scarring of back-skin wounds. The authors elucidated that aging induced the suppression of keratinocyte-derived SDF1 as the underlying mechanism. This idea is further corroborated by enhanced tissue regeneration in young skin in response to genetic deletion of SDF-1 [91].

### 4.2. Wound-Induced Hair Follicle Neogenesis (WIHN)

In laboratory mice (*Mus musculus*), small (<1 × 1 cm^2^) excisional wounds heal with scars and with permanent loss of hair follicles, whereas large (>1.5 × 1.5 cm^2^) full-thickness wounds may culminate in wound-induced hair follicle neogenesis (WIHN) in the central wound area. The process of de novo hair follicle formation seen in large wounds resembles hair follicle embryonic development, typically 14–19 days after wounding [92].

**Figure 1:**
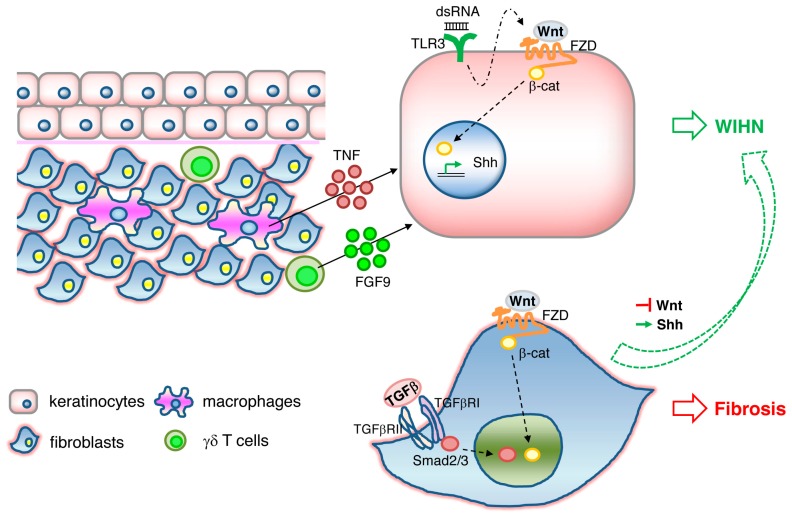
Signaling pathways involved in wound-induced hair follicle neogenesis (WIHN). Wnt/β-catenin signaling and downstream Shh signaling in keratinocytes activates WIHN. TNF released from macrophages, FGF9 released from γδ T cells, and double-strand RNA binding to TLR3 in keratinocytes induces WIHN. Wnt//β-catenin and TGFβ/Smad signaling in fibroblasts results in fibrosis. Activation of Shh or suppression of Wnt pathways in fibrosis is sufficient to redirect Wnt-active wound fibroblasts to WIHN.

WIHN is dependent on epidermal Wnt/β-catenin signaling. WIHN can also be promoted by direct modulation of Wnt signaling components such as Wnt ligands Wnt7a [92] and Wls [93], or indirectly through its negative regulator CXXC-type zinc finger protein 5 (CXXC5) [94], or through an external stimulus triggering Wnt activation, such as FGF9 released from dermal γδ T cells [95] or TNF released from wound-infiltrating macrophages [96]. In addition, it has been shown that WIHN requires transient epithelial Msx2 expression [97] and can be inhibited by prostaglandin D2 through the receptor Gpr44 [98]. A scheme illustrating the signaling pathways triggering WIHN is depicted in Figure 1.

While Wnt/β-catenin activity plays a critical role in epidermal stem cell maintenance, hair follicle development and regeneration, it is also well known to have opposite effects in fibroblasts, where Wnt signaling appears to mediate skin fibrosis and pathological scarring [99,100,101,102]. TLR3 expression on keratinocytes and activation of the downstream IL-6/STAT3 pathway is necessary for WIHN to occur, possibly through the promotion of transcription of Wnt/β-catenin signaling and Shh/Gli2 signaling [103]. In contrast, prolonged activation of IL6/STA3 in fibroblasts leads to fibrosis [104] and aggravated scarring such as keloids [105]. It is therefore tempting to speculate that TLR3/IL-6/STAT3, one of the damage-associated molecular pattern (DAMP) pathways, acts upstream of Wnt/β-catenin activity (Figure 1). A recent study gives greater insight into this dichotomous effect of Wnt activity on scarring and regeneration. Lim and colleagues showed that activation of the Shh pathway in the dermis is both necessary and sufficient for WIHN [106]. Shh activation converts Wnt-active wound fibroblasts into dermal papilla and thereby redirects fibrotic programs back to regeneration (Figure 1). Therefore, the activation of the signaling cascades TLR3, Wnt, and Shh in either keratinocytes or fibroblasts determines the outcome of WIHN or fibrosis. How these signals are spatially and temporally fine-tuned to orchestrate regeneration or scarring remains elusive.

It is worth noting that WIHN does not fully result in complete regeneration of skin, as occurs in lower vertebrates. For example, the newly formed hairs are not pigmented, and the melanocyte niche is not re-established [92]. The stimulation of WIHN by overexpression of Shh or Wnt does not diminish the extent of collagen deposition and overall ECM composition of the wounds [106]. Rather, the newly formed hairs are imbedded in fibrotic scar tissue and restricted to wound centers alone [107]. Moreover, WIHN is not observed in skin wounds that are inflicted in humans or even in rats [108]. Nevertheless, the strategies we have learnt from the WIHN model that inhibit Wnt/β-catenin signaling specifically in the dermis or boost Wnt/β-catenin signaling specifically in the epidermis may lead to new and effective antiscarring therapeutics.

### 4.3. Scarless Repair of the Oral Mucosa

Injuries to the oral mucosa heal with very minimal scars as compared to back-skin injuries. This diversity of scarless versus scarring across these two anatomic skin locations is an outcome of distinct fibroblast lineages that reside in each skin compartment. Oral cavity fibroblasts are derived from the neural crest, and these lineages are intrinsically different from back-skin fibroblast lineages [13]. We previously purified neural crest fibroblasts from the oral cavity and found intrinsic differences between these Wnt1 lineage positive fibroblasts (WPFs) and the EPFs from back-skin. Transplantation of purified WPFs into back-skin wounds generated very diffuse stromal compartments with very minimal scars, whereas transplanting purified EPFs into the oral cavity generated ectopic scars that mimic back-skin scars. These reciprocal transplantation experiments indicate that fibroblast lineages retain intrinsic fibrotic potentials that are unaltered by their local environment [13], and that scarring versus scarless outcomes across anatomic skin locations are outcomes of diverse fibroblast lineages with cell-intrinsic differences.

By using paired and sequential biopsies of human oral and cutaneous wounds, Iglesias-Bartolome and colleagues recently showed that wound-activated transcriptional networks are present in the unwounded state in oral mucosa. Compared to skin keratinocytes, the higher expression of transcriptional regulators SOX2 (sex-determining region Y-box 2) and PITX1 (paired-like homeodomain 1) in oral keratinocytes is associated with higher migratory capacity for faster re-epithelialization [109].

### 4.4. Scarless Skin Regeneration in African Spiny Mice (Genus Acomys)

Skin regeneration after injury has been observed in some mammalian species such as African spiny mice (*Acomys kempi*, *Acomys percivali*, *Acomys cahirinus*) [110]. *Acomys* can heal full-thickness skin wounds in a scar-free manner with complete regeneration of epidermal appendages such as hair follicles and sebaceous glands, erector pili muscles, adipocytes, and panniculus carnosus muscle. The regenerative ability of *Acomys* has been documented in the wild following predation-induced skin autotomy, and also experimentally in the lab by performing full-thickness excisional wounds in back-skin [110].

So far, most studies on *Acomys* skin regeneration are at a descriptive level. *Acomys* skin shows substantially low tensile strength [111], a thinner dermis layer but thicker layers of adipose tissue and panniculus carnosus muscle [111,112], a blunted immune system with a distinct lack of macrophages [111], and longer but less frequent hair follicle cycles all in a synchronous manner [112]. In an ear wounding model, Simkin and colleagues showed that the reactive oxygen species (ROS) production was surprisingly stronger and persisted longer during regeneration in *Acomys*. Macrophages which are required for regeneration initiation are significantly reduced in *Acomys* [113]. Further, the transcriptional regulation of wound healing genes is altered in *Acomys*, including upregulation of the collagen triple helix repeat containing 1 (Cthrc1) gene, MMPs, tenascin C and N, fibronectin, and aggrecan, and downregulation of TIMPs [111]. These gene signatures indicate that in *Acomys*, wound healing is regulated differently, which may favor a pro-regenerative outcome.

## 5. Summary and Perspectives

Skin wound repair is a multifaceted process that aims to accomplish two major tasks: restoring the barrier functions of the skin so as to prevent further blood loss and infection, and restoring the physiological and mechanical properties. Scarring and regeneration can be viewed as extreme restoration of one versus the other: patching wounds with dense plugs of ECM makes for quick sealing of wounds, whereas regeneration takes longer but culminates in functional restoration. Healed wounds are therefore a compromise of the bifurcation of fibrosis and regeneration, and skin repair is carried out on a spectrum from overhealing, as in hypertrophic scars, keloids, and scleroderma, to regeneration, as in fetal wound healing, oral mucosal repair, and WIHN. Understanding of the mechanism underlying the balance of scarring and regeneration is the key for future manipulation towards scarless regeneration in a fully controlled manner. 

**Figure 2:**
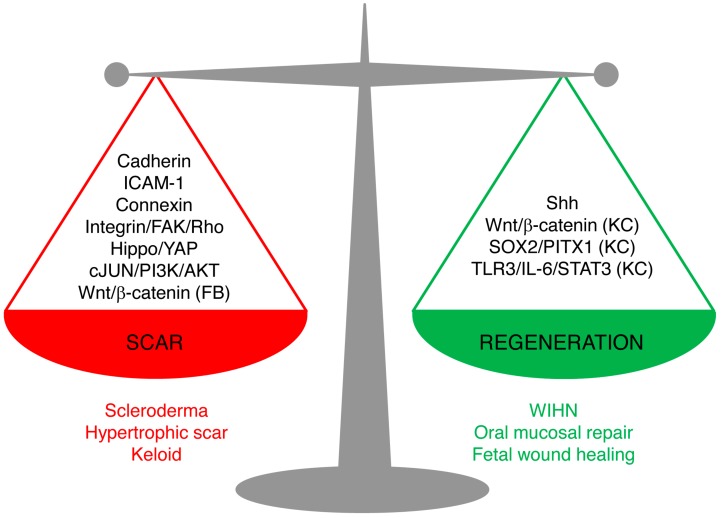
Signaling pathways leading to scarring or regeneration. The outcome of skin wound healing is a balance of signaling pathways leading to scarring or regeneration. Upregulation of Cadherins, ICAM-1, or Connexins or overactivation of Hippo/YAP, integrin/FAK/Rho GTPase, c-JUN/PI3K/AKT, or Wnt/β-catenin signaling in fibroblasts leads to pathological scarring, such as scleroderma, hypertrophic scar, and keloids. Activation of Sonic Hedgehog (Shh) signaling in keratinocytes and wound fibroblasts, or activation of Wnt/β-catenin, SOX2/PITX1, or TLR3/IL-6/STAT3 in keratinocytes (but not in fibroblasts) promotes regeneration, as seen in wound-induced hair follicle regeneration, oral mucosa repair, and fetal scarless healing. (FB), only in fibroblasts; (KC), only in keratinocytes.

The new studies reviewed here shed light on the cellular and molecular mechanisms leading to scarring and regeneration upon skin injury. The key signaling pathways are summarized in Figure 2. We believe that these pathways are not isolated, but rather interconnected. Hence, the triggers of the fibrotic or regenerative process likely require cross-talk of multiple pathways. Among them, the signal cascades involving cell–matrix adhesion and cell–cell adhesion between fibroblasts emerge as important candidates for future research on scarring mechanisms.

Despite fast progress in the field in recent years, the governing mechanism as an integrated network is far from being understood. New state-of-the-art techniques such as lineage tracing, intravital microscopy, and single-cell transcriptional and epigenetic profiling, coupled with ECM composition and migration analysis, are expected to continually illuminate the underlying mechanisms of skin scarring, and may pave the way to future promising pro-regenerative approaches for skin injuries.

## Figures and Tables

**Table 1:** Dermal fibroblast subsets with intrinsic fibrotic property.

Fibrotic Subset	Nonfibrotic Subset	Species	Reference
En1 lineage^+^	En1 lineage^−^	mouse	[13,14]
Prrx1 enhancer^+^	Prrx1 enhancer^−^	mouse	[15]
ADAM12^+^		mouse	[16]
nuclear c-JUN^+^		human	[17]
Dlk1^+^	Lrig1^+^	mouse (E16.5)	[12,18]
CD36^+^CD90^+^	CD39^+^CD90^+^	human	[19]
FAP^-^CD90^+^	FAP^+^CD90^−^	human	[20]
Sca1^+^CD34^+^CD29+		mouse	[21]
CD26^+^		mouse	[13,22]

## Abbreviations

ADAM12	A disintegrin and metalloprotease domain 12
APs	Adipocyte precursor cells
Dlk1	Delta like non-canonical Notch ligand 1
ENFs	Engrailed-1 lineage negative fibroblasts
EPFs	Engrailed-1 lineage positive fibroblasts
FAK	Focal adhesion kinase
FAP	Fibroblast activation protein
GAGs	Glycosaminoglycans
Lrig1	Leucine-rich repeats and immunoglobulin-like domains protein 1
PDGF	Platelet-derived growth factor
Prrx1	Paired-related homeobox 1
Shh	Sonic Hedgehog
ST2	Suppression of tumorigenicity 2
TAZ	Transcriptional coactivator with PDZ-binding motif
TGFβ	Transforming growth factor beta
TIMP	Tissue inhibitor of metalloproteinases
WIHN	Wound-induced hair follicle neogenesis
YAP	Yes-associated protein
